# Polycystic Horseshoe Kidney: A Rare Coexistence as a Challenge for the Surgeons. Case Report

**DOI:** 10.15388/Amed.2022.29.2.7

**Published:** 2022-06-29

**Authors:** Dionysios Prevezanos, Nikolaos Garmpis, Dimitrios Dimitroulis, Anna Garmpi, Vasiliki E. Georgakopoulou, Christos Damaskos

**Affiliations:** Renal Transplantation Unit, Laiko General Hospital, Athens, Greece; Second Department of Propedeutic Surgery, Laiko General Hospital, Medical School, National and Kapodistrian University of Athens, Athens, Greece; N.S. Christeas Laboratory of Experimental Surgery and Surgical Research, Medical School, National and Kapodistrian University of Athens, Athens, Greece; Second Department of Propedeutic Surgery, Laiko General Hospital, Medical School, National and Kapodistrian University of Athens, Athens, Greece; First Department of Propedeutic Internal Medicine, Laiko General Hospital, Medical School, National and Kapodistrian University of Athens, Athens, Greece; Department of Pulmonology, Laiko General Hospital, Athens, Greece; Renal Transplantation Unit, Laiko General Hospital, Athens, Greece; N.S. Christeas Laboratory of Experimental Surgery and Surgical Research, Medical School, National and Kapodistrian University of Athens, Athens, Greece

**Keywords:** polycystic kidney, horseshoe, nephrectomy

## Abstract

Autosomal dominant polycystic kidney disease (ADPKD) with concomitant horseshoe kidney is an extremely rare entity. In this case, we report a 45-year-old male patient with ADPKD and a horseshoe kidney who demonstrated hypertension, urological complications, and discomfort symptoms such as pain, breathing difficulties, and abdominal meteorism. After preoperative assessment and planning, the patient underwent nephrectomy. Bilateral nephrectomy without dividing the isthmus was performed successfully. The isthmus, which had complicated vasculature and was full of cysts, remained intact, avoiding severe bleeding and infection. The postoperative course was uneventful. Keeping the isthmus intact in such cases is a challenge for the surgeon. The rarity of polycystic horseshoe kidney in combination with the altered abdominal anatomy requires the proper preoperative strategy in order to avoid intraoperative complications.

## Santrauka.

Autosominiu dominantiniu būdu paveldima inkstų policistozė (angliškai trumpinama ADPKD) kartu su pasagos inkstu yra itin retas reiškinys. Čia pateikiamu atveju pacientui, 45 metų vyrui, kuriam yra autosominiu dominantiniu būdu paveldima inkstų policistozė bei pasagos inkstas, buvo padidėjęs kraujospūdis, urologinės komplikacijos bei nemalonios savijautos simptomai – skausmas, kvėpavimo sunkumas ir abdominalinis meteorizmas. Įvertinus paciento būklę iki operacijos ir suplanavus operaciją, jam buvo atlikta nefrektomija. Sėkmingai atlikta abipusė nefrektomija nepadalijant sąsmaukos tarp inkstų. Sąsmauka, kurioje buvo sudėtinga kraujagyslių sistema ir daug cistų, operuojant nebuvo paliesta, taip išvengta stipraus kraujavimo ir infekcijos. Tokiu atveju nepaliesti sąsmaukos yra iššūkis chirurgui. Pooperacinis laikotarpis praėjo be problemų. Situacijos, kai yra policistinis pasagos inkstas ir netipinė pilvo anatomija, retos, todėl reikia tinkamos priešoperacinės strategijos, kad būtų išvengta komplikacijų operacijos metu.

## Introduction

During embryogenesis, several mutations or false expressions of the genes can cause a variety of diseases that lead to surgical conditions. Horseshoe kidney is a common congenital anatomic malformation in which, mainly, the lower poles of both kidneys are fused in a thick tissue called an isthmus. Polycystic kidney disease (PKD) is a less common hereditary autosomal disease caused by mutations in the PKD1 (85%) or PKD2 (15%) genes. Consequently, end-stage renal failure is, in most cases, inevitable [1]. The prevalence of gene mutations in PKD1 is approximately 1 in 474 and varies between 1 in 400 and 1 in 4000 in PKD2 [1, 2].Thus, polycystic horseshoe kidney is a very rare disease, that has been described in less than 30 reports in the global literature, with a prevalence of 1 in 134,000 to 1 in 8,000,000 [3]. To date, there is no report that genetically correlates the two diseases. Indications for surgical intervention include making space within the iliac fossa for renal transplantation, proteinuria, infection, symptoms from mass effect, urolithiasis, or tumors [4]. As far as surgical intervention is concerned, both laparoscopic and open techniques have been described without clear benefit of one over the other, due to the extremely rare incidence. Indeed, laparoscopic nephrectomy is technically challenging due to the renal size. However, it is not technically possible to perform laparoscopic bilateral nephrectomy without dividing the isthmus. By doing so, severe bleeding, cystic rupture, and abscesses can be caused.

Hence, we report a case of a male patient with a polycystic horseshoe kidney who underwent open bilateral nephrectomy without dividing the isthmus and avoiding further complications.

## Case presentation

**Figure 1.**
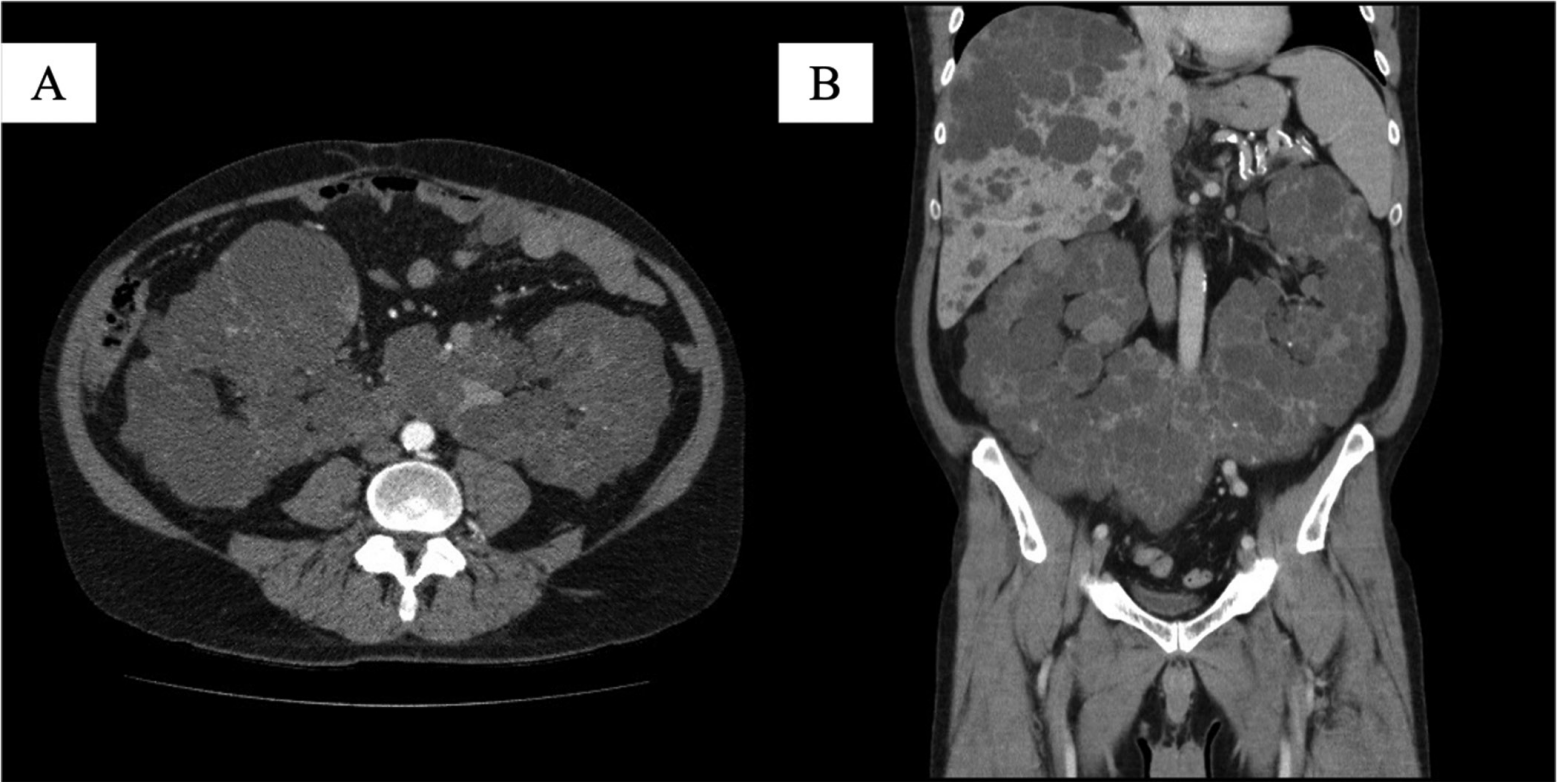
Computed tomography (CT) showing renal fusion and massive cysts. A: Axial section; B: Coronal section.

**Figure 2.**
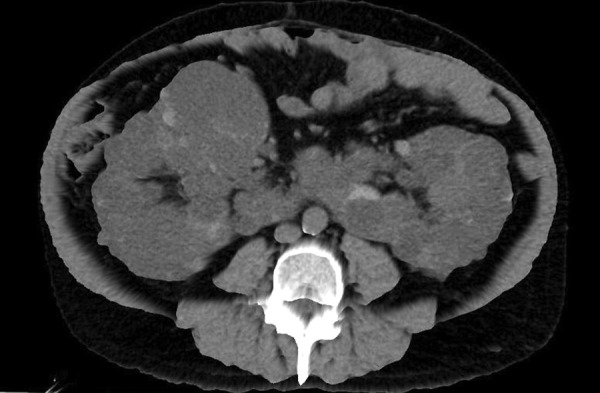
3D reconstruction of computed tomography image showing renal fusion and massive cysts; axial section.

**Figure 3.**
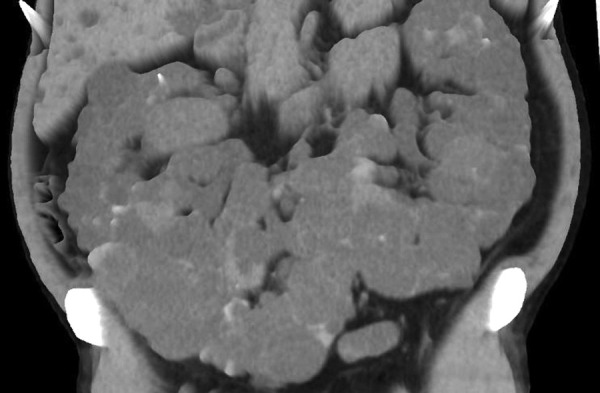
3D reconstruction of computed tomography image showing renal fusion and massive cysts; coronal section.

A 49-year-old male patient was admitted to our department for elective bilateral kidney nephrectomy due to ADPKD alongside a horseshoe kidney. The initial diagnosis took place 35 years ago when the patient underwent a random radiological examination with abdominal ultrasound (US). At that time, the patient was asymptomatic, and it was suggested that he change his lifestyle habits (diet, intense physical exercise). Eight years later, the patient referred to symptoms such as pain, breathing problems, hematuria, urinary tract infections, and meteorism of the abdomen. A follow-up US demonstrated nephrolithiasis. In addition, he developed hypertension, which was controlled with medication, and 6 years later, started hemodialysis because of renal failure. From that point on, the symptoms were getting worse, and last year, it was proposed to perform a new radiological examination. Computed tomography (CT) confirmed the existence of a polycystic horseshoe kidney and its progression through the years and demonstrated a challenging altered anatomy (Figures 1, 2 and 3). It was decided to perform a surgical excision.

In the operating theater, the patient was placed in a supine position and a long midline abdominal incision was performed. After entering the peritoneal cavity, the right colon was mobilized along the whole length of the line of Toldt and the right kidney was dissected by the retroperitoneal space. The right ureter was identified and cut with clips. The upper pole was also mobilized with the preservation of the right adrenal gland. The renal hilum was then discovered. Two right renal arteries and two right renal veins were identified, and all of them were stapled and transected separately using a vascular linear stapler. The same procedures were also performed on the left side. The left adrenal gland was preserved, and there were two left renal arteries and two left renal veins on this side too. The whole polycystic horseshoe kidney was removed from the abdomen without dividing its isthmus. Two negative pressure drainage systems were put in the left and right iliac fossa. The total operative time was two and a half hours, and the amount of blood lost was about 250 mL. No blood transfusion was needed. The patient was successfully transferred to the clinic, and he was discharged one week later without any complications. Renal transplantation has not yet been performed.

**Figure 4.**
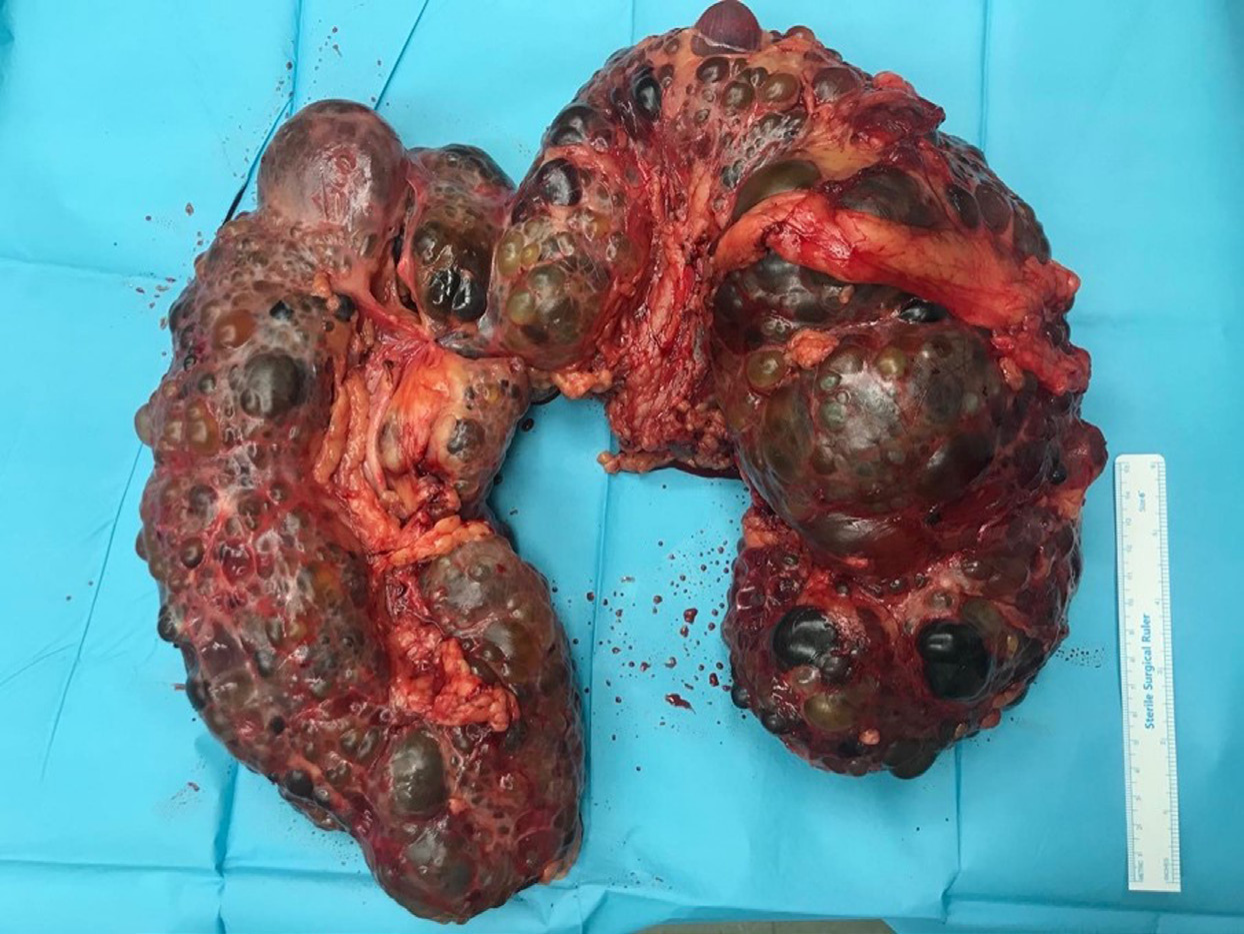
Macroscopic view of the surgical specimen of a polycystic horseshoe kidney with intact isthmus.

The weight of the surgical specimen was 4600 g, with a length of 30 cm and a width of 24 cm. Moreover, the length of the right lobe was 30 cm and the length of the left was 28 cm, while the width was 13 cm and 14 cm, respectively (Figure 4). Histological evaluation confirmed the diagnosis of PKD, as it was described with multiple cystic lesions; while the renal parenchyma was atrophic and replaced by fibrous connective tissue.

Our patient gave written informed consent to participate in the current study.

## Discussion

Considering the rarity of the concomitant polycystic and horseshoe kidney, there is no sufficient consensus for this disease. In 1965, Trapp *et al*. reported the first documented case of nephrectomy for polycystic horseshoe kidney [5]. Through the literature, fewer than thirty cases have been reported since then.

In 2009, Glodny *et al*. described the isthmus as a completely bloody tissue with anatomical variants. In 185 cases, there was an average of 2.4 right renal arteries, 1.9 left renal arteries, 2.4 right renal veins, and 1.7 left renal veins [2]. These findings indicate that any separation of the two kidneys can lead to uncontrollable bleeding. In addition, any possible rupture of the cysts can create a fertile ground for peritonitis.

Both open and laparoscopic techniques (pure or hand-assisted) have been reported. Dason *et al*. have described a purely laparoscopic technique in which unilateral nephrectomy was performed in order for future transplantation to take place. To avoid bleeding, seven distinct renal vessels were identified and clipped, ensuring that the isthmus consists of renal parenchyma and complicated anatomy [6]. Additionally, Hammontree *et al*. have reported a patient who underwent hand-assisted laparoscopic bilateral nephrectomy for the same purpose [7]. Neither of these authors did mention any further symptoms preoperatively, in contrast with our patient, who had hypertension, urologic complications (hematuria, urinary tract infections), pain, breathing problems, and abdominal meteorism.

A vast majority of the cases presented in the global literature were treated with open surgery either with lumbar or midline incision, with division of the isthmus, and were admitted to the intensive care unit (ICU) post-operatively. Yao *et al*. presented a febrile female patient with a polycystic horseshoe kidney who underwent an isthmus undivided bilateral nephrectomy due to severe renal infection. The length and width of the surgical sample were 20.3 cm and 14.2 cm, respectively, showing that it is possible to preserve the isthmus while avoiding the intra-operative complications. The patient was transferred to the ICU for 2 days and started hemodialysis after 2 days [8]. In comparison, our patient had multiple episodes of hematuria and urinary tract infections. The size of his kidney was determined to be 30x24 cm by US and CT preoperative imaging assessment. As a result, open intervention became unavoidable. Keeping the isthmus intact saved a lot of surgical time as well as limited the risk of bleeding and possible infection of the peritoneal cavity. The patient was transferred to the clinic immediately and not to the ICU, and started hemodialysis the day after.

This extremely rare entity refers to two concomitant different diseases: the horseshoe kidney and the polycystic kidney. In a third of the cases, the horseshoe kidney is asymptomatic, while the rest is predisposed to urological sequalae due to obstruction, such as nephrolithiasis, hydronephrosis, infection, and vesicoureteral reflux. The surgical approach consists of division of the fused isthmus and generally is not recommended due to the increased risk of complications such as infections, fistulas, leakages, and bleeding [9]. Regarding, though, the polycystic kidney, about 8–10% of the patients develop end-stage renal disease while others have complications like rupture of the cysts, bleeding, and potential peritonitis, making surgical intervention/transplantation inevitable [10]. Prior to intervention, adequate review of preoperative imaging and awareness of the difficult anatomy that will be encountered are critical in this operation. Thus, the surgeon is obligated to deal with double trouble and choose the proper technique. Due to the rarity of the polycystic horseshoe kidney, our knowledge is limited, and further studies are needed in order to elucidate the surgical technique and its outcome.

## Conclusion

In conclusion, the combination of these two different diseases makes the surgical technique more challenging, as the isthmus should be preserved and this preservation along with the fast-track rehabilitation are still in preliminary stages.
